# REGGAE: a novel approach for the identification of key transcriptional regulators

**DOI:** 10.1093/bioinformatics/bty372

**Published:** 2018-05-07

**Authors:** Tim Kehl, Lara Schneider, Kathrin Kattler, Daniel Stöckel, Jenny Wegert, Nico Gerstner, Nicole Ludwig, Ute Distler, Markus Schick, Ulrich Keller, Stefan Tenzer, Manfred Gessler, Jörn Walter, Andreas Keller, Norbert Graf, Eckart Meese, Hans-Peter Lenhof

**Affiliations:** 1 Center for Bioinformatics, Saarland Informatics Campus, Saarland University, Saarbrücken D-66041, Germany; 2 Department of Genetics, Saarland University, Saarbrücken D-66041, Germany; 3 Theodor-Boveri-Institute/Biocenter, Developmental Biochemistry, and Comprehensive Cancer Center Mainfranken, Würzburg University, Würzburg, Germany; 4 Department of Human Genetics, Medical School, Saarland University, Homburg, Germany; 5 Institute for Immunology, Johannes Gutenberg University Mainz, Mainz, Germany; 6 Department of Internal Medicine III, School of Medicine, Technische Universität München, Munich, Germany; 7 German Cancer Consortium (DKTK), German Cancer Research Center (DKFZ), Heidelberg, Germany; 8 Department of Pediatric Oncology and Hematology, Medical School, Saarland University, Homburg, Germany

## Abstract

**Motivation:**

Transcriptional regulators play a major role in most biological processes. Alterations in their activities are associated with a variety of diseases and in particular with tumor development and progression. Hence, it is important to assess the effects of deregulated regulators on pathological processes.

**Results:**

Here, we present REGulator-Gene Association Enrichment (REGGAE), a novel method for the identification of key transcriptional regulators that have a significant effect on the expression of a given set of genes, e.g. genes that are differentially expressed between two sample groups. REGGAE uses a Kolmogorov–Smirnov-like test statistic that implicitly combines associations between regulators and their target genes with an enrichment approach to prioritize the influence of transcriptional regulators. We evaluated our method in two different application scenarios, which demonstrate that REGGAE is well suited for uncovering the influence of transcriptional regulators and is a valuable tool for the elucidation of complex regulatory mechanisms.

**Availability and implementation:**

REGGAE is freely available at https://regulatortrail.bioinf.uni-sb.de.

**Supplementary information:**

Supplementary data are available at *Bioinformatics* online.

## 1 Introduction

The transcriptional program in eukaryotic cells is controlled by transcriptional regulators like transcription factors, coregulators and epigenetic modifiers. Hence, transcriptional regulators play a major role in most biological processes (Vaquerizas *et al.*, 2009) and alterations in their activities have been associated with a variety of diseases (Lee and Young, 2013). For instance, mutations in many genes involved in congenital heart disease are known to be transcriptional regulators, e.g. NKX2-5, GATA4 and TBX5 (Papavassiliou and Papavassiliou, 2016; McCulley and Black, 2012). Deregulated transcriptional regulators are also associated with neurodegenerative diseases, for example heat shock factor 1 with Alzheimer’s, Huntington’s and Parkinson’s disease (Neef *et al.*, 2011). In cancer, many transcriptional regulators are known to be involved in tumor development and progression (Darnell, 2002; Nebert, 2002; Papavassiliou and Papavassiliou, 2016). For example, steroid receptors like the estrogen receptor ESR1 are involved in breast cancer (Robinson *et al.*, 2013) or the androgen receptor in prostate cancer (Yuan *et al.*, 2014). The central roles of transcriptional regulators in many diseases and their potential to regulate a large number of target genes make transcriptional regulators putative candidates for novel drug targets (Bhagwat and Vakoc, 2015; Yeh *et al.*, 2013).

The advent of high-throughput sequencing technologies made it possible to identify binding sites for a large number of regulators, using e.g. ChIP-Seq experiments. This technological progress motivated the development of novel methods for assessing the influence of transcriptional regulators. A subclass of these algorithms uses over-representation analysis to detect transcription factors that have more targets in a list of deregulated genes than expected by chance. Essaghir *et al.* implemented TFactS (Essaghir *et al.*, 2010), a web server that adopts the hypergeometric test. Yang *et al.* developed an R-package, called *DCGL* (Yang *et al.*, 2013a), that offers two statistical tests: (i) *TED* applies a binomial probability model to test whether targets of a certain regulator are enriched in a list of deregulated genes and (ii) *TDD* computes the density of deregulated genes in the targets of a certain regulator. Alternative approaches are based on correlation coefficients to identify associations between regulators and target genes. *RIF1* and *RIF2* (Reverter *et al.*, 2010) combine the correlations between a regulator and its targets with the degrees of differential expression of the targets. Another correlation-based approach, called *Correlation Set Analysis* (Huang *et al.*, 2012), investigates the effect of regulators on disease populations using the mean correlation of all target pairs per regulator. Gonçalves *et al.* proposed a network-based approach to prioritize regulators, called *TFRank* (Goncalves *et al.*, 2011). Poos *et al.* provided an R package (*MIPRIP*) that applies a machine learning approach, based on mixed integer linear programming, which predicts important regulatory interactions influencing a single gene (Poos *et al.*, 2016). Kawakami *et al.* presented a weighted *t*-test *wPGSA* (Kawakami *et al.*, 2016), which incorporates the probability of regulation in the considered ChIP-Seq experiments. Furthermore, Gonçalves *et al.* developed *Regulatory Snapshots* (Gonçalves *et al.*, 2012), a web server for the identification of important regulatory modules using time series gene expression data. A systematic evaluation of some of these approaches was conducted by Yu *et al.* (2014). A comprehensive description of all used methods can be found in Supplementary Material S1.

Here, we introduce an alternative approach for the identification of influential transcriptional regulators, called *REGulator-Gene Association Enrichment* (REGGAE) analysis. REGGAE combines association scores between regulators and their target genes with non-parametric enrichment analysis to prioritize the influence of the considered regulators. We implemented REGGAE as part of the GeneTrail2 C++ library (Stöckel *et al.*, 2016) as well as the RegulatorTrail web service (Kehl *et al.*, 2017), which can be freely accessed at https://regulatortrail.bioinf.uni-sb.de.

To demonstrate the capabilities of our approach, we tested REGGAE and related algorithms in two different application scenarios. First, we compared estrogen receptor positive (ER+) and estrogen receptor negative (ER−) breast cancer cell lines to reveal the key regulators primarily responsible for the phenotypic differences between the two classes. Second, we analyzed perturbation signatures of (i) mouse lymphomas with artificially induced overexpression of MYC and (ii) knock-out experiments of NANOG, POU5F1 and SOX2 in human embryonic stem cells to examine if the different methods are able to identify the perturbed regulators. The conducted experiments demonstrate that REGGAE excels in revealing the most influential transcriptional regulators and hence may be a valuable tool for the elucidation of complex regulatory mechanisms.

## 2 Materials and methods

Here, we introduce REGGAE our new algorithm for the identification of transcriptional regulators that have a significant influence on a given set of differentially expressed target genes and we describe the databases used in our application scenarios.

### 2.1 Regulator–target gene interactions (RTIs)

In order to identify influential regulators, REGGAE relies on a predefined list of regulator–target gene interactions (RTIs). Here, an RTI is defined as a pair (regulator, target gene), where the regulator has an experimentally determined binding site in a regulatory region of the target gene (e.g. promotor or enhancer). For the RegulatorTrail web service (Kehl *et al.*, 2017), we have recently built an extensive collection of RTIs based on external databases. To this end, we have combined information originating from seven databases: ChEA (Lachmann *et al.*, 2010), ChIP-Atlas (chip-atlas.org), ChipBase (Yang *et al.*, 2013b), ENCODE (Sloan *et al.*, 2016), JASPAR (Mathelier *et al.*, 2016), SignaLink (Fazekas *et al.*, 2013) and TRANSFAC (Matys, 2003). For our analyses, we have used the entire collection of RTIs (Version 2) for humans and mice. For more information about the content of our RTI database, processing steps and provenance data, please refer to the respective RegulatorTrail documentation page (https://regulatortrail.bioinf.uni-sb.de/help?topic=rtis).

### 2.2 Regulator-gene association enrichment analysis

In this section, we describe our REGGAE algorithm, which is based on a combination of non-parametric enrichment analysis and association scores between regulators and their target genes. A standard input for a REGGAE analysis consists of (i) a normalized gene expression matrix, where the m samples (columns) belong to two groups, e.g. disease and control samples and (ii) a collection of RTIs.

Based on the gene expression matrix and the collection of RTIs, REGGAE estimates the influence of transcriptional regulators by performing the following steps:

#### 2.2.1 Step 1: Calculating differentially expressed genes

REGGAE offers a variety of methods to calculate genes that are differentially expressed between the two groups: (log-)fold-changes, correlation coefficients, signal-to-noise ratio, *z*-test, *f*-test, a variety of *t*-tests and several rank-sum tests. For count data, we additionally provide DESeq2 (Love *et al.*, 2014), edgeR (Robinson *et al.*, 2010) and RUVSeq (Risso *et al.*, 2014). Users can select one of these methods to calculate all genes that are either up- or down-regulated and sort the resulting gene lists according to their test values. To simplify matters, we consider in the following only one of the two (up- or down-regulated) sorted gene lists: $D=\{g_{1},g_{2},\ldots ,g_{n}\}$.

#### 2.2.2 Step 2: Calculating the influence of regulators for every deregulated gene

**Fig. 1.**
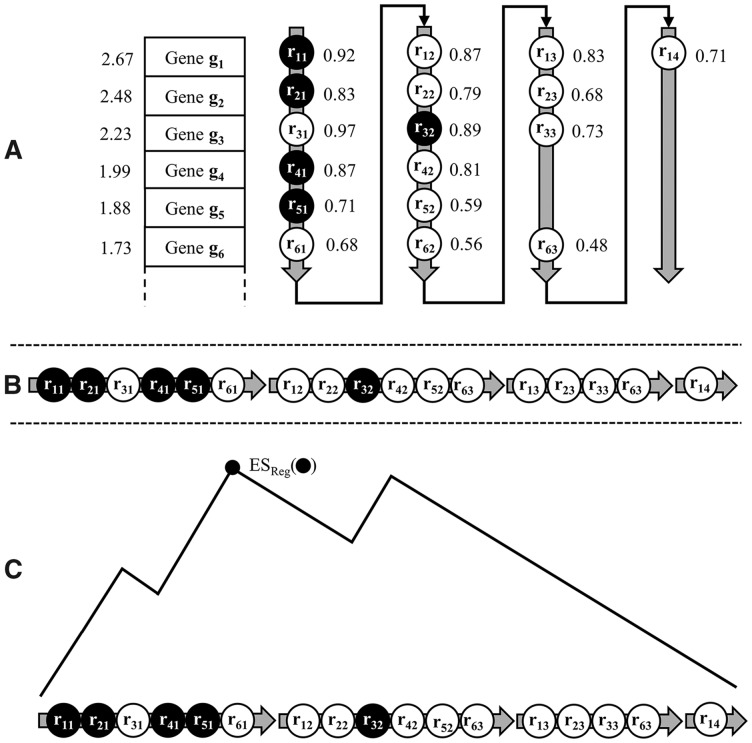
REGGAE workflow. (A) Overexpressed genes $g_{1},\;g_{2},\;\ldots ,\;g_{n}$ (second column) are sorted according to their *t*-scores (first column). For each gene $g_{i}$, the list of regulators $\left\{r_{i1},r_{i2},\;\ldots \right\}$ is sorted with respect to the absolute values of the corresponding correlation coefficients. The black nodes represent a selected regulator that controls five target genes. (B) Shows the new regulator list L created by sorting the elements column by column. (C) Enrichment analysis (running sum) for the transcriptional regulator marked in black

For each deregulated gene $g_{i}\in D$, the given collection of RTIs contains a list of regulators $R_{g_{i}}\;=\left\{r_{i1},r_{i2},\ldots |\}\right\}$ that may influence the expression of $g_{i}$. For every regulator–target pair, we calculate the correlation between the two variables (expression values) across all samples using either Pearson’s correlation coefficient (Pearson, 1895) for linear dependencies or Spearman’s rank correlation coefficient (Spearman, 1961) for non-linear dependencies. We then sort the regulator list $R_{g_{i}\;}$for each gene $g_{i}$ with respect to the (absolute or signed) values of the correlation coefficients (cf. Fig. 1A).

#### 2.2.3 Step 3: Creating the sorted regulator list

Based on a sorted list $D=\{g_{1},g_{2},\ldots ,g_{n}\}$ of genes and their regulator lists $R_{g_{i}}=\{r_{i1},r_{i2},\ldots \}$, we create a new list $L=\{r_{11},r_{21},\ldots ,r_{n1},r_{12},r_{22},\ldots \}$ that sorts the involved regulators column by column as shown in Figure 1A and B.

#### 2.2.4 Step 4: Enrichment analysis

Since regulators with a high impact should be enriched at the top of the list L, we carry out an enrichment analysis on L for each regulator in the RTI collection by using either the Wilcoxon rank-sum (WRS) test (Wilcoxon, 1945) or the unweighted version of the Kolmogorov–Smirnov (KS) test (Keller *et al.*, 2007; Subramanian *et al.*, 2005) (cf. Fig. 1C). The resulting *P*-values are adjusted using the Benjamini and Yekutieli method (Benjamini and Yekutieli, 2001). Finally, all regulators are sorted with respect to their *P*-values.

Technical noise in gene expression measurements might have an influence on the calculated correlation coefficients and subsequently on the order of the regulators. To account for this, we carry out the following bootstrapping (Efron, 1979) scheme to improve the robustness of the method:

- Create B bootstrap samples, where each sample is a gene expression matrix generated by randomly selecting m columns from the original gene expression matrix with replacements.
- Repeat steps 2–4 for each bootstrap sample.
- Assign the median *P*-value as the new score for each regulator.

The bootstrap samples can also be used to estimate standard deviations, mean absolute deviations and confidence intervals. For the latter, we implemented a method to compute bias-corrected and accelerated bootstrap intervals (Efron, 1987).

Additionally, we suggest not only to perform one REGGAE analysis using the lists of significantly deregulated genes, but also to vary the number of considered genes and to repeat the analysis for gene lists of different lengths. The respective result lists can then be aggregated. In our framework, we provide implementations for rank- as well as *P*-value aggregations.

## 3 Results

To evaluate the performance of REGGAE and alternative approaches, we considered two different application scenarios. First, we compared ER+ and ER− breast cancer cell lines to uncover key regulators associated with the ER. Second, we analyzed perturbation signatures of (i) mouse lymphomas with artificially induced MYC overexpression and (ii) knock-out experiments of NANOG, POU5F1 and SOX2 in human embryonic stem cells. In both perturbation studies, we examined if the different methods are able to identify the perturbed regulator.

### 3.1 ER-positive breast cancer cells

Breast cancer is one of the most common types of cancer and the second leading cause of cancer death among women (Siegel *et al.*, 2017). One of the clinically most relevant breast cancer subtypes are ER+ tumors, which comprise around 70% of diagnosed cases (Fillmore *et al.*, 2010) and generally have a better prognosis than ER− tumors (Bae *et al.*, 2015). ER+ tumors are usually treated using endocrine therapy (Lumachi *et al.*, 2013). This therapy may include drugs that compete with estrogen for the ER (e.g. tamoxifen) or aromatase inhibitors that prevent estrogen production from precursor molecules (Smith and Dowsett, 2003), the latter especially administered in post-menopausal women (Mokbel *et al.*, 2006).

Here, we applied REGGAE to analyze the breast cancer dataset published by Heiser *et al.* (Heiser *et al.*, 2012). The dataset contains gene expression profiles of 37 breast cancer cell lines, for which we obtained the status of the ER from a study by Neve *et al.* (2006) (cf. Supplementary Material S2). In total, we compared 16 ER+ and 21 ER− cell lines to find transcriptional regulators that have a strong influence on gene expression differences between the two groups.

To this end, we used the shrinkage *t*-test (Opgen-Rhein and Strimmer, 2007) to calculate for each gene a *t*-score mirroring the expression differences between the two groups (ER+ versus ER− samples) and sorted all genes with respect to their *t*-scores. From the resulting list, we selected all genes that are significantly up-regulated (P < 0.01) in ER+ tumors (1719), as well as the top 250, 500, 750 and 1000 genes. We applied REGGAE to all five lists and aggregated the respective result lists using the sum of all ranks and the maximum of the five *P*-values. The aggregated *P*-values were adjusted using the method proposed by Benjamini and Yekutieli (Benjamini and Yekutieli, 2001). Parameters for all analyses and corresponding results can be found in Supplementary Materials S3 and S4, respectively.

#### 3.1.1 Robustness

**Fig. 2.**
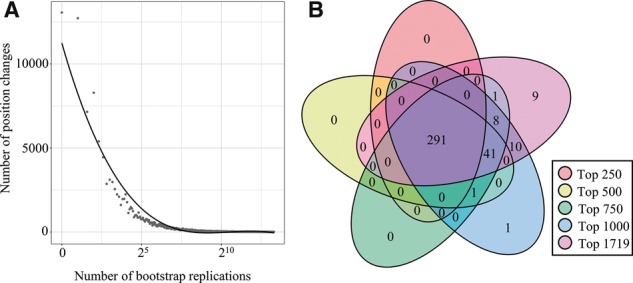
Robustness of REGGAE results. (A) Effect of an increasing number of bootstrap replications on the order of regulators in the REGGAE result lists for up-regulated genes. The number of bootstrap samples (x-axis) is plotted against the total number of position changes (y-axis). (B) Venn diagram depicting the overlap of REGGAE results for the five different input lists

First, we analyzed the effect of bootstrapping on the five REGGAE result lists of up-regulated genes of length 250, 500, 750, 1000 and 1719 (significantly up-regulated). To this end, we checked after each bootstrap iteration how many regulators changed their position compared to the previous iteration. This was done by calculating the total number of regulator pairs (a,b) that swap their order, i.e. if $r_{i}\left(a\right)<\;r_{i}(b)$ and $r_{i+1}\left(a\right)>\;r_{i+1}(b)$ or vice versa, where $r_{i}(a)$ is the rank of regulator a in iteration i. The results for the list of length 1000, shown in Figure 2A, illustrate that with an increasing number of bootstrap replications, the number of fluctuating regulator pairs converges, until only a handful regulators pairs swap their position with ‘equally important’ neighbors. The results for lists of lengths 250, 500, 750 and 1719 can be found in Supplementary Material S5.

Additionally, we calculated the overlaps for the different lists and generated a Venn diagram depicting the corresponding overlaps (cf. Fig. 2B). The figure shows that the result lists are highly stable. With an increasing test set size, the total number of significant results increases slightly, but seems to converge when more genes are considered. The largest increase (42 new significant regulators) has been observed when transitioning from 250 to 500 genes.

#### 3.1.2 Comparison to other methods

In order to compare REGGAE with alternative methods, we applied all available approaches with the exception of *MIPRIP* and *wPSGA* to the breast cancer dataset. *MIPRIP* can only predict the effects of all regulators on a single target gene and hence was not applied. The *wPSGA* method could not be used as information about the number of ChIP-Seq experiments that confirm an RTI cannot be reliably deduced from the integrated databases. All methods were tested using our RTI collection and the same input datasets. A complete list of the used parameters and results of all methods can be found in Supplementary Materials S3 and S4. Runtimes for all methods are depicted in Table 1.

**Table 1.** Runtime comparison for top 250 up-regulated genes

Method	Runtime (s)
CSA^a^	450.27 (±78.76)
REGGAE^b^	174.98 (±1.69)
REGGAE (without bootstrapping)	23.40 (±0.36)
RIF1	23.60 (±0.28)
RIF2	23.85 (±0.10)
TDD	14.86 (±0.63)
TED	658.20 (±29.80)
TFactS	42.37 (±0.23)
TFRank	116.74 (±4.22)

*Note*: Runtimes were obtained on an Intel Core i7-3770 processor.

a CSA analysis was conducted using 1 000 000 permutations.

b REGGAE analysis was performed using 1000 bootstrap replications.

Please note that a major part of the computation time of REGGAE (without bootstrapping) is spent on reading-in the large RTI database, which is only carried out once during the initialization of the procedure.

**Fig. 3.**
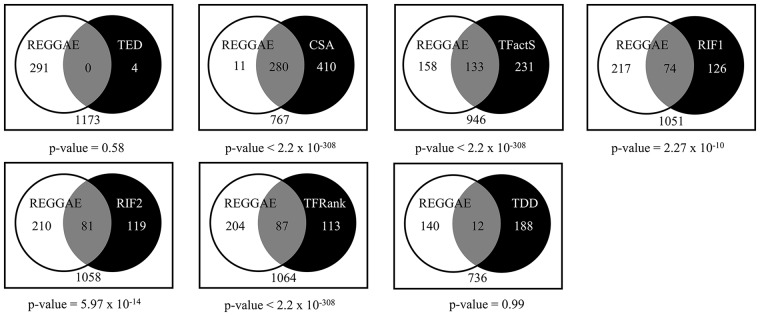
Venn diagrams showing the overlap of the different methods with the list generated by REGGAE. All results were calculated based on the aggregated lists of the most up-regulated genes. *P*-values for the overlaps were calculated using the hypergeometric test

Since most of the available methods are based on statistical tests with different null hypotheses, any comparison of their results must be interpreted with utmost caution. Nevertheless, we calculated the overlaps between REGGAE and the alternative approaches. To this end, we selected all significant results after FDR-adjustment for methods that provided *P*-values (REGGAE, TFactS, CSA and TED) and the top 200 regulators for all other approaches (RIF1, RIF2, TFRank and TDD) (cf. Fig. 3). The comparison showed that the REGGAE result list has significant overlaps with five out of the seven tested approaches.

While there are strong overlaps between REGGAE and most alternative methods, the actual rankings of the different approaches differ extremely. Table 2 shows the top five regulators identified by REGGAE for up-regulated genes (columns 1 and 2) and if these genes have also been detected by the other methods. The columns of Table 2 show either corresponding *P*-values or scores if no *P*-values are provided and the ranks of the genes in the result lists. All top five REGGAE candidates have also been identified by CSA and TFRank as significant. Notably, with respect to the rankings of the top candidates, REGGAE and TFRank yield very similar results that differ strongly from the remaining methods. TFactS detected 2 of the top 5 regulators as significant, RIF1 and RIF2 detected 4 out of the 5 among their top 200 candidates. In the following section, we will discuss the top five regulators identified by REGGAE and we will provide some evidence that the prioritization of REGGAE and TFRank is biologically meaningful.

**Table 2.** Top five regulators identified by REGGAE in comparison to other approaches

Regulators	REGGAE	CSA	RIF1	RIF2	TDD	TED	TFactS	TFRank
FOXA1	$6.34\times 10^{-141}(1)$	$9.76\times 10^{-6}(359)$	-2.87 (116)	8.34 (18)	$8.4\times 10^{-6}(956)$	1.0 (843)	1.0 (953)	6.92 (2)
GATA3	$3.23\times 10^{-137}(2)$	$9.76\times 10^{-6}(421)$	-2.73 (113)	5.16 (62)	$8.7\times 10^{-6}(747)$	1.0 (681)	0.05 (369)	6.56 (3)
ESR1	$6.52\times 10^{-129}$(3)	$9.76\times 10^{-6}(509)$	-1.93 (229)	−0.10 (915)	$8.4\times 10^{-6}(949)$	1.0 (440)	1.0 (790)	10.28 (1)
MYB	$6.34\times 10^{-125}(4)$	$9.76\times 10^{-6}(262)$	-2.07 (130)	4.14 (75)	8.4$\times 10^{-6}(878)$	1.0 (606)	0.31 (519)	5.45 (6)
SPDEF	$2.60\times 10^{-118}$(5)	$9.76\times 10^{-6}(40)$	-3.05 (32)	8.54 (15)	1.4$\times 10^{-5}(434)$	1.0 (892)	3.6×10^−19^(72)	6.44 (4)

*Note*: For REGGAE, CSA and TFactS adjusted *P*-values are depicted. For RIF1, RIF2 and TFRank, which do not provide *P*-values, the respective test statistic value is shown. Numbers in parentheses represent the rank in the sorted result list.

#### 3.1.3 Influential regulators

The top five regulators identified by REGGAE are FOXA1, GATA3, ESR1, MYB, and SPDEF. All five have already been described as prognostic markers in breast cancer, which positively correlate with a favorable outcome of the disease (Mehra *et al.*, 2005; Mehta *et al.*, 2012; van 't Veer *et al.*, 2002; West *et al.*, 2001). Of those, FOXA1, ESR1 and GATA3 are not only reported as co-expressed (Sachs *et al.*, 2013) and co-localized (Kong *et al.*, 2011) in breast cancer cells, but there is even strong evidence suggesting they might form an enhanceosome that regulates many genes involved in the ER signaling cascade (Kong *et al.*, 2011). Furthermore, FOXA1, GATA3, ESR1 and SPDEF are reported as master regulators in FGFR2 signaling and breast cancer risk in ER+ cells (Fletcher *et al.*, 2013). Notably, only TFRank and REGGAE ranked these important regulators of ER+ breast cancer cells as the top candidates.

We also assessed the top regulators of the other methods. The results show that, while all methods were able to identify breast cancer relevant regulators, only RIF1 and RIF2 identified regulators with direct connections to ER+ breast cancer. RIF1 detected LRIG1, a gene that is known to correlate with relapse-free survival in ERα-positive breast cancer (Krig *et al.*, 2011). RIF2 identified MAP3K1, a regulator for which a single nucleotide polymorphism (rs88912) is associated with poor prognosis of hormone receptor positive tumors (Kuo *et al.*, 2017), as well as GRHL1, a downstream target of ESR1 (Zheng *et al.*, 2016). A detailed discussion of the results can be found in Supplementary Material S6.

### 3.2 Perturbation signatures

Perturbation signatures are predestined to study the effect of transcriptional regulators. While gene knock-outs can be utilized to simulate loss-of-function mutations (LoF), artificially induced overexpression mimics activating genetic alterations. In both cases, resulting gene expression changes allow investigating the influence of the perturbed regulators on the transcriptomic level.

Here, we compared gene expression profiles of artificially induced overexpression of MYC in lymphomas of *Eµ-Myc*-transgenic mice with those of wild-type lymph node samples. We also investigated the effects of knock-out experiments of NANOG, POU5F1 (OCT4) and SOX2 in human embryonic stem cells with respect to a set of controls. For both cases, we examined whether the different methods could retrace the effects of the perturbed transcription factors and thus identify them as the key regulators.

#### 3.2.1 MYC-induced lymphoma cells

The MYC proto-oncogene is a transcription factor that is involved in the control of cell growth, division and metabolism, affecting the transcription of a plethora of target genes (Dang, 2012; Meyer and Penn, 2008).

In many cancer types, MYC overexpression is associated with aggressive disease and alterations in MYC expression levels play an essential role in tumor development and progression. The *Eµ-Myc* mouse model resembles B cell specific MYC activation by coupling the *Myc* oncogene to the immunoglobulin enhancer. Emerging B cell lymphomas are characterized by high MYC levels and this model is widely used to study the mechanisms of MYC-driven lymphomagenesis (Boxer and Dang, 2001; Harris, 1988).

Here, we compared the gene expression of a set of 50 B cell lymphomas from Eµ-myc-transgenic mice with 10 mouse wild-type lymph node samples from GEO (GSE7897) (Mori *et al.*, 2008) using a shrinkage *t*-test (Opgen-Rhein and Strimmer, 2007). We selected the 250 most up- and down-regulated genes and then applied all methods for the identification of key regulators using the collection of mouse RTIs. Parameters for all analyses and corresponding results can be found in Supplementary Materials S6 and S7. The respective ranks of MYC in the sorted result lists generated by the various methods are shown in Table 3A.

**Table 3.** Results for perturbation experiments of (A) artificially induced overexpression of MYC in *Eµ-Myc* mice (B) knock-out experiments of the pluripotency factors NANOG, POU5F1 and SOX2

	A	B		
Method	MYC	NANOG	POU5F1	SOX2
CSA	**281** | **126**	**574** | 571	510 | **273**	510 | **259**
REGGAE	**1** | **1**	**1** | **91**	**1** | **1**	**6** | **4**
RIF1	**126** | **186**	791 | **148**	795 | **171**	285 | 555
RIF2	**8** | 251	**144** | **193**	762 | **190**	332 | **34**
TDD	466 | 492	815 | 771	822 | 800	467 | 523
TED	208 | 225	567 | 501	683 | 588	682 | 682
TFactS	**404** | 528	**318** | **308**	**531** | **319**	**170** | **99**
TFRank	**1** | **3**	**113** | **2**	**200** | **1**	499 | **1**

*Note*: For all methods ranks in the sorted result lists for up- and down-regulated genes are shown (up | down). Ranks are highlighted in **bold** if corresponding *P*-values are statistically significant for methods that provide *P*-values (CSA, REGGAE, TED and TFactS) or are amongst the top 200 genes for all other methods (RIF1, RIF2, TDD and TFRank).

The results show that CSA, REGGAE, RIF1 and TFRank were able to identify MYC as relevant based on both input lists. RIF2 and TFactS detected MYC only for up-regulated genes. Although most methods were able to connect MYC to the perturbed gene expression, only REGGAE and TFRank were able to identify the proto-oncogene as the most important regulator.

Besides that, REGGAE was able to identify various other transcription factors and co-factors regulated by MYC (cf. Supplementary Material S8). Most prominently (rank 2 for up-regulated genes), the histone acetyltransferase KAT2A, which is up-regulated by MYC to influence global chromatin structure and alter gene expression (Knoepfler *et al.*, 2006). Next to that, REGGAE identifies two E2F transcription factors, which are known to play essential roles in oncogenic MYC signaling (Leone *et al.*, 2001; Rempel *et al.*, 2009). Finally, the two MYC hallmark genes RAD23B and TRIM28 are also among the TOP25 regulators.

Taken together, this underscores REGGAE's ability to not only identify central activators, but also to identify downstream effectors of these regulators.

#### 3.2.2 Knock-out of pluripotency factors

NANOG, POU5F1 (OCT4) and SOX2 are fundamental regulators in embryonic stem cells (ESCs). They maintain pluripotency, regulate self-renewal and control cell fate determination (Loh *et al.*, 2006).

In this analysis, we used knock-out experiments of each pluripotency factor in human embryonic stem cells (GSE34921) (Wang *et al.*, 2012) to check if the different methods are able to identify the effect of the perturbed regulator. To this end, we compared gene expression profiles of the respective perturbation signatures and corresponding controls using a shrinkage *t*-test (Opgen-Rhein and Strimmer, 2007). For each list, we selected the 250 most up- and down-regulated genes and then applied all methods to evaluate their performance. Parameters for all analyses and corresponding results can be found in Supplementary Materials S9 and S10. The ranks of the perturbed regulators are shown in Table 3B.

A comparison of the results shows that REGGAE and TFactS identified the perturbed regulators in all result lists as significant, TFRank in five out of six, RIF2 in four, CSA in three, RIF2 in two. In terms of prioritization, we again see that REGGAE and TFRank outperform alternative methods. REGGAE was able to find the perturbed regulator in five of the six cases as one of the top candidates and TFRank in three cases.

## 4 Application to Wilms tumors

Besides the analyses presented in Section 3, we also applied REGGAE to gene expression profiles of 33 biopsies of Wilms tumor (WT), which is a childhood nephroblastoma.

The goal was to elucidate pathogenic mechanisms that contribute to a WT histopathological subtype, which is characterized by predominant blastemal tissue and associated with an elevated malignancy. Applying REGGAE to a set of genes deregulated in blastemal WTs revealed that regulators involved in embryonic development and epigenetic processes like chromatin remodeling and histone modification play an essential role in blastemal WTs. In particular, we identified TCF3 as the central regulatory element in this context and provided evidence for its role as master regulator of blastemal WTs. Results for this use case will be presented in a separate manuscript (Kehl *et al.*, submitted for publication).

## 5 Discussion

We present a novel approach for the identification and prioritization of transcriptional regulators that have a strong influence on the expression of a given set of genes. Our method complements the repertoire of existing approaches with an alternative that prioritizes transcriptional regulators with a KS-like test statistic and implicitly combines correlation with enrichment analysis. REGGAE excels in the prioritization of the regulators by incorporating both the positions of target genes in the analyzed gene list and the influence of the regulators on each gene.

In Step 2 of the REGGAE algorithm, we utilize correlation coefficients to sort all regulators. The power of these correlation coefficients is restricted by the used sample size. Although we allow users to perform REGGAE analyses with small sample sizes, we recommend using at least 10 samples that should ideally be evenly distributed among the two groups. For the computation of the correlation coefficients, REGGAE offers the methods proposed by Pearson and Spearman. Since linear models are commonly used to model regulatory interactions between genes, we selected Pearson’s correlation coefficient as default option. If, however, users assume a non-linear relationship between a regulator and its target genes, Spearman’s correlation coefficient should be used instead. Additionally, there are alternative methods that could also be applied to sort the regulators. For example, the MIPRIP package could be used to estimate the effect of each regulator. Alternatively, TEPIC (Schmidt *et al.*, 2017) could be applied to calculate affinity scores of transcription factors, if open chromatin regions are available.

In our application scenarios, we used the WRS test in Step 4 of the REGGAE algorithm. We additionally performed all analyses using the KS test instead. Corresponding results can be found in Supplementary Materials S4, S8 and S10. The KS test performed similarly to the WRS test, but the latter provided better rankings.

We also recommend the combination of REGGAE results for input lists of different sizes. Although this is an optional step that increases the runtime, we are convinced that the aggregation of the different results provides more stable rankings. Comparisons of the different result lists allow users to oppose the different ranking of the top candidates and to assess their stability.

A limitation of all approaches for regulator effect analysis is that the results of each analysis depend on the quantity and quality of available datasets of RTIs, which mainly stem from ChIP-Seq experiments of certain cell types. Here, a regulator is assigned to its target gene if it binds within a predefined interval around the transcription start site. Depending on the size of this window, the considered region can also contain enhancer regions. Although it has been shown that the binding of transcription factors to regulatory regions, like enhancers, often strongly affects the gene expression of the ‘nearest’ genes, see e.g. (Ernst *et al.*, 2011), this assignment is still a simplified approach that can lead to false assignments. In the future, the assignments of regulators to target genes could potentially be improved by incorporating chromosome conformation capturing techniques like Hi-C, see e.g. (González *et al.*, 2015). Another problem is that, while the DNA binding of some regulators has been extensively studied, binding information for some regulators is still missing or only available for other species like mouse or rat. Furthermore, binding information is often only available for certain cell types, however, not for the investigated cell type. To solve this problem, we have integrated the binding information of all available ChIP-Seq experiments for each regulator, irrespective of cell type, but specific for each organism. This can, of course, lead to false positive and false negative interactions. However, we assume that a small number of faulty interactions will only have a moderate effect on the REGGAE results.

We used REGGAE as well as seven alternative approaches (CSA, RIF1, RIF2, TDD, TED, TFactS and TFRank) in two application scenarios to evaluate their performance. First, we compared ER+ and ER− breast cancer cell lines. Here, our results indicate that most methods find highly overlapping results, however, with substantially different rankings. Although most methods were able to assign at least some of the central regulators of ER+ cells as being relevant, REGGAE and TFRank excelled in terms of the actual ranking of those regulators.

Second, we analyzed perturbation signatures of artificially induced overexpression of MYC in lymphomas of *Eµ-Myc*-transgenic mice as well as knock-out experiments of NANOG, POU5F1 and SOX2 in human embryonic stem cells. In both cases, we tested if the different approaches are able to identify the perturbed regulators. A comparison of the results showed that in most cases only REGGAE and TFRank could identify the perturbed regulators as top candidates in the respective result lists. Although, most methods were able to detect the influence of at least some perturbed regulators, only REGGAE could successfully identify all four perturbagens as significant in all cases.

The two application scenarios show that REGGAE and TFRank outperform all other methods in terms of the regulator prioritization. A reason for this might be that both methods integratively analyze the effects of all regulators and do not just assess them separately. However, it is noteworthy that while both methods generally work well, REGGAE clearly outperforms TFRank for up-regulated target genes in all knock-out experiments.

Moreover, REGGAE provides information that facilitates the interpretability of the results. This is on the one hand achieved by keeping track of the mean signed correlation of each regulator and all considered target genes to estimate whether the regulator acts as activator or repressor. On the other hand, we provide several measures of confidence like *P*-values, confidence intervals and standard deviations that allow to judge the significance and validity of each result.

Results of both scenarios demonstrated that REGGAE is well suited for uncovering the influence of transcriptional regulators and might even aid in the detection of novel biomarkers. Consequently, REGGAE may also be a valuable tool for the elucidation of complex pathogenic mechanisms in other diseases.

## Funding

This work was supported by Deutsche Forschungsgemeinschaft [LE952/3-2] and Deutsche Krebshilfe [111944, 50-2709-Gr2].

*Conflict of Interest*: none declared.

## Supplementary Material

Supplementary Data
