# Supplementary material for: REGGAE: a novel approach for the identification of key transcriptional regulators
Source: Bioinformatics. 2018 May 7;34(20):3503–10. doi: 10.1093/bioinformatics/bty372 (PMC6184769; doi:10.1093/bioinformatics/bty372)
Supplement: Supplementary Data [file bty372_suppl_data.zip › Supplement_S1_Bioinformatics.docx]

**REGGAE: a novel approach for the identification of key transcriptional regulators**

Tim Kehl^1,*^, Lara Schneider^1^, Kathrin Kattler^2^, Daniel Stöckel^1^, Jenny Wegert^3^, Nico Gerstner^1^, Nicole Ludwig^4^, Ute Distler^5^, Markus Schick^7^, Ulrich Keller^7,8^, Stefan Tenzer^5^, Manfred Gessler^3^, Jörn Walter^2^, Andreas Keller^1^, Norbert Graf^6^, Eckart Meese^4^, Hans-Peter Lenhof^1^

^1^Center for Bioinformatics, Saarland Informatics Campus, Saarland University, Saarbrücken, Germany, ^2^Department of Genetics, Saarland University, Saarbrücken, Germany, ^3^Theodor-Boveri-Institute/Biocenter, Developmental Biochemistry, and Comprehensive Cancer Center Mainfranken, Würzburg University, Würzburg, Germany, ^4^Human Genetics, Saarland University, Homburg, Germany, ^5^Institute for Immunology, Johannes Gutenberg University Mainz, Mainz, Germany, ^6^Department of Pediatric Oncology and Hematology, Medical School, Saarland University, Homburg, Germany, ^7^Internal Medicine III, School of Medicine, Technische Universität München, Munich, Germany, ^8^German Cancer Consortium (DKTK), German Cancer Research Center (DKFZ), Heidelberg, Germany

*To whom correspondence should be addressed.

# Supplement S1

In this section, we provide an overview of the methods used in this manuscript. Table 1 provides a description of the general approach as well as the used statistical test or algorithm. Table 2 provides an overview of the required input, produced output and several features that might distinguish the different approaches.

**Table 1. Description of the objective, general approach and underlying algorithms for the different methods discussed in the main manuscript.**

| Method | Objective | Approach | Statistical test / Algorithm |
| --- | --- | --- | --- |
| CSA | CSA examines if all targets of a regulator exhibit a high pairwise co-expression. | CSA uses a statistical test that calculates the mean of all pairwise correlation coefficients between all targets of each analyzed regulator. The significance of the results is computed using a permutation test. | -Correlation coefficient |
| REGGAE | REGGAE identifies and prioritizes transcriptional regulators with a significant effect on deregulated target genes. | REGGAE prioritizes transcriptional regulators with a Kolmogorov-Smirnov-like test statistic and implicitly combines correlation with enrichment analysis. | -Differential gene expression  -Correlation coefficient  -Enrichment analysis (Wilcoxon rank-sum test, GSEA) |
| RIF1 and RIF2 | RIF1 and RIF2 examine if a regulator is differentially co-expressed between the two sample groups with respect to its target genes. | Both metrics integrate the change in correlation between regulator and the differentially expressed (DE) target genes, the amount of differential expression as well as the abundance of DE genes. According to the authors, a " RIF analysis assigns an extreme score to those TF that are consistently most differentially co-expressed with the highly abundant and highly DE genes (RIF1), and to those TF with the most altered ability to predict the abundance of DE genes (RIF2) " (Reverter,A. *et al., 2010*). | -Differential gene expression  -Correlation coefficients  -Abundance of genes (raw gene expression) |
| TDD | TDD calculates the density of deregulated target pairs in comparison to all targets of the analyzed regulator. | TDD computes the ratio of deregulated target genes compared to all target genes of the analyzed regulator. | -Enrichment analysis (Density) |
| TED | TED examines if a certain regulator has more targets in the list of deregulated target genes than expected by chance. | TED uses a binomial test to check for each regulator if the used RTI database has more targets in the analyzed test set than expected by chance. | -Enrichment analysis (ORA - Binomial test) |
| TFactS | TFactS examines if a certain regulator has more targets in the list of deregulated target genes than expected by chance. | TFactS uses a hypergeometric test to check for each regulator if the used RTI database has more targets in the analyzed test set than expected by chance. | -Enrichment analysis (ORA - Hypergeometric test) |
| TFRank | TFRank prioritizes regulators involved in a process of interest. | TFRank uses a network-based approach to propagate the weights (scores) of differentially expressed genes to targeting regulators over the complete network of RTIs in order to find the regulators that might be responsible gene expression changes based on the used network structure. | -Differential gene expression  -Network based approach |

**Table 2. General overview of the required input, output and distinguishing properties of the different methods discussed in the main manuscript.**

| Method | Required input | Output | Ability to estimate if the regulator acts as an activator or repressor | Measures of confidence | Simultaneous assessment of regulator effects* |
| --- | --- | --- | --- | --- | --- |
| CSA | Gene-expression matrix,  Score list (test set) | P-value (estimated based on permutation tests) | No | -P-values | No |
| REGGAE | Gene-expression matrix,  Score list (test set) | P-value (exact) | Yes | -P-values  -Confidence intervals  -Standard deviation | Yes |
| RIF1 and RIF2 | Gene-expression matrix | Score | Yes |  | No |
| TDD | Gene set (no weights) | Density | No |  | No |
| TED | Gene set (no weights) | P-value (exact) | No | P-values | No |
| TFactS | Gene set (no weights) | P-value (exact) | No | P-values | No |
| TFRank | Score list | Score | No |  | Yes |

*REGGAE and TFRank are the only methods that do not assess the influence of each regulator individually but in relation to all other regulators.

# References

Essaghir,A. *et al.* (2010) Transcription factor regulation can be accurately predicted from the presence of target gene signatures in microarray gene expression data. *Nucleic Acids Research*, **38**, e120–e120.

Goncalves,J.P. *et al.* (2011) TFRank: network-based prioritization of regulatory associations underlying transcriptional responses. *Bioinformatics*, **27**, 3149–3157.

Huang,C.-L. *et al.* (2012) Correlation set analysis: detecting active regulators in disease populations using prior causal knowledge. *BMC Bioinformatics*, **13**, 46.

Kehl,T. *et al.* (2017) RegulatorTrail: a web service for the identification of key transcriptional regulators. *Nucleic Acids Research*, **45**, W146–W153

Reverter,A. *et al.* (2010) Regulatory impact factors: unraveling the transcriptional regulation of complex traits from expression data. *Bioinformatics*, **26**, 896–904.

Yang,J. *et al.* (2013) DCGL v2.0: An R Package for Unveiling Differential Regulation from Differential Co-expression. *PLOS ONE*, **8**, e79729.

Yu,H. *et al.* (2014) Algorithms for network-based identification of differential regulators from transcriptome data: a systematic evaluation. *Science China Life Sciences*, **57**, 1090–1102.
