# Supplementary material for: REGGAE: a novel approach for the identification of key transcriptional regulators
Source: Bioinformatics. 2018 May 7;34(20):3503–10. doi: 10.1093/bioinformatics/bty372 (PMC6184769; doi:10.1093/bioinformatics/bty372)
Supplement: Supplementary Data [file bty372_suppl_data.zip › Supplement_S2_Bioinformatics.docx]

**REGGAE: a novel approach for the identification of key transcriptional regulators**

Tim Kehl^1,*^, Lara Schneider^1^, Kathrin Kattler^2^, Daniel Stöckel^1^, Jenny Wegert^3^, Nico Gerstner^1^, Nicole Ludwig^4^, Ute Distler^5^, Markus Schick^7^, Ulrich Keller^7,8^, Stefan Tenzer^5^, Manfred Gessler^3^, Jörn Walter^2^, Andreas Keller^1^, Norbert Graf^6^, Eckart Meese^4^, Hans-Peter Lenhof^1^

^1^Center for Bioinformatics, Saarland Informatics Campus, Saarland University, Saarbrücken, Germany, ^2^Department of Genetics, Saarland University, Saarbrücken, Germany, ^3^Theodor-Boveri-Institute/Biocenter, Developmental Biochemistry, and Comprehensive Cancer Center Mainfranken, Würzburg University, Würzburg, Germany, ^4^Human Genetics, Saarland University, Homburg, Germany, ^5^Institute for Immunology, Johannes Gutenberg University Mainz, Mainz, Germany, ^6^Department of Pediatric Oncology and Hematology, Medical School, Saarland University, Homburg, Germany, ^7^Internal Medicine III, School of Medicine, Technische Universität München, Munich, Germany, ^8^German Cancer Consortium (DKTK), German Cancer Research Center (DKFZ), Heidelberg, Germany

*To whom correspondence should be addressed.

# Supplement S2

In this section, we describe the breast cancer dataset published by Heiser et al. (Heiser *et al.*, 2012). The dataset contains gene expression profiles of 46 breast cancer cell lines. We obtained the status of the estrogen-receptor (ER) for each cell line from a study by Neve et al. (2006). After the assignment, we obtained 5 distinct sample groups:

## Group 1: Estrogen-receptor positive (ER+):

600MPE, BT474, BT483, CAMA1, HCC1428, LY2, MCF7, MDAMB134VI, MDAMB175VII, MDAMB361, MDAMB415, T47D, UACC812, ZR751, ZR7530, ZR75B

## Group 2: Estrogen-receptor negative (ER-):

AU565, BT20, BT549, HCC38, HCC70, HCC202, HCC1143, HCC1187, HCC1937, HCC1954, HCC2185, HCC3153, HS578T, MCF10A, MCF12A, MDAMB157, MDAMB231, MDAMB453, SKBR3, SUM225CWN, SUM1315MO2

## Group 3: Presumably estrogen-receptor positive (ER[+]):

SUM52PE

## Group 4: Presumably estrogen-receptor negative (ER[-]):

SUM149PT, SUM159PT

## Group 5: No information available (NA):

184B5, HCC1395, HCC1419, HCC1806, MCF10F, SUM185PE

In all analyses in Section 3.1, we compared ER+ (Group 1) and ER- cells lines (Group 2).

# References

Neve, R.M. *et al.* (2006) A collection of breast cancer cell lines for the study of functionally distinct cancer subtypes. *Cancer Cell.* **10(6)**, 515-27*.*

Heiser, L.M. *et al.* (2012) Subtype and pathway specific responses to anticancer compounds in breast cancer. *Proceedings of the National Academy of Sciences*, **109**, 2724–2729.
