# Supplementary material for: REGGAE: a novel approach for the identification of key transcriptional regulators
Source: Bioinformatics. 2018 May 7;34(20):3503–10. doi: 10.1093/bioinformatics/bty372 (PMC6184769; doi:10.1093/bioinformatics/bty372)
Supplement: Supplementary Data [file bty372_suppl_data.zip › Supplement_S5_Bioinformatics.docx]

**REGGAE: a novel approach for the identification of key transcriptional regulators**

Tim Kehl^1,*^, Lara Schneider^1^, Kathrin Kattler^2^, Daniel Stöckel^1^, Jenny Wegert^3^, Nico Gerstner^1^, Nicole Ludwig^4^, Ute Distler^5^, Markus Schick^7^, Ulrich Keller^7,8^, Stefan Tenzer^5^, Manfred Gessler^3^, Jörn Walter^2^, Andreas Keller^1^, Norbert Graf^6^, Eckart Meese^4^, Hans-Peter Lenhof^1^

^1^Center for Bioinformatics, Saarland Informatics Campus, Saarland University, Saarbrücken, Germany, ^2^Department of Genetics, Saarland University, Saarbrücken, Germany, ^3^Theodor-Boveri-Institute/Biocenter, Developmental Biochemistry, and Comprehensive Cancer Center Mainfranken, Würzburg University, Würzburg, Germany, ^4^Human Genetics, Saarland University, Homburg, Germany, ^5^Institute for Immunology, Johannes Gutenberg University Mainz, Mainz, Germany, ^6^Department of Pediatric Oncology and Hematology, Medical School, Saarland University, Homburg, Germany, ^7^Internal Medicine III, School of Medicine, Technische Universität München, Munich, Germany, ^8^German Cancer Consortium (DKTK), German Cancer Research Center (DKFZ), Heidelberg, Germany

*To whom correspondence should be addressed.

# Supplement S5


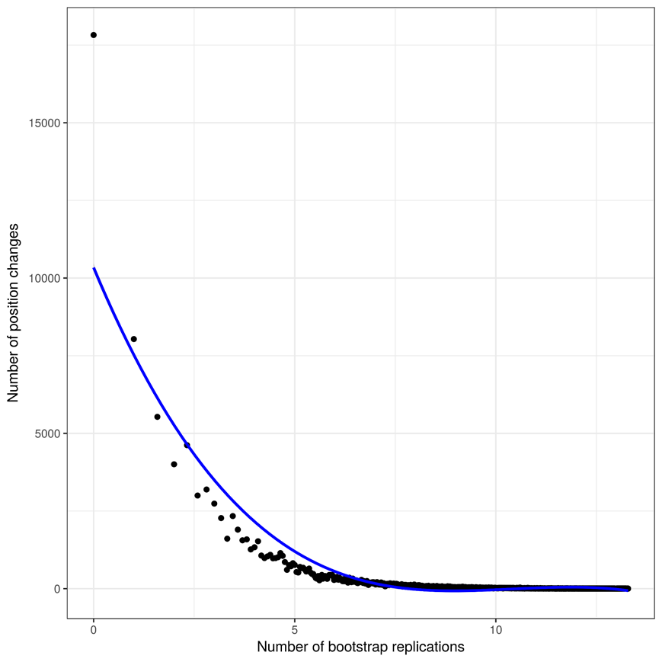

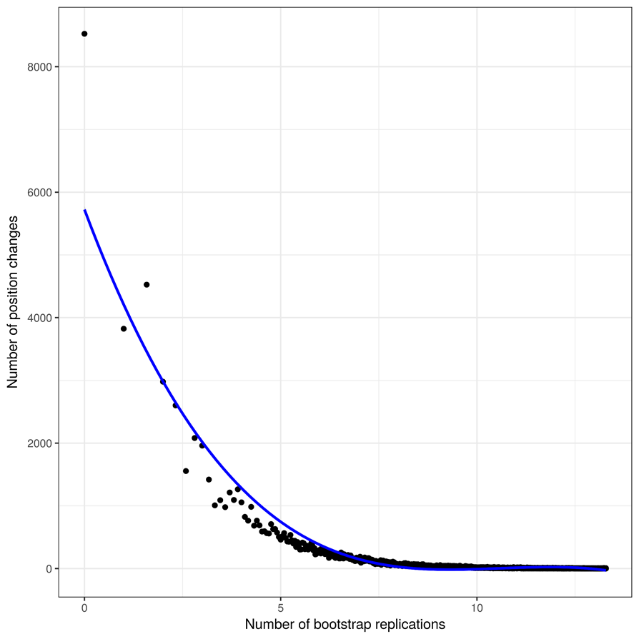
In this section, we provide additional results for Section 3.1.1 in the main manuscript. The following plots depict how the robustness of the REGGAE results increasing with the number of conducted bootstrapping replications.

Figure 2. Effect of an increasing number of bootstrap replications on the order of the regulators in the REGGAE result lists for the top 500 upregulated genes. The number of bootstrap samples (x-axis) is plotted against the total number of position changes (y-axis). (B) Venn diagram depicting the overlap of REGGAE results for the five different input lists.

Figure 1. Effect of an increasing number of bootstrap replications on the order of the regulators in the REGGAE result lists for the top 250 upregulated genes. The number of bootstrap samples (x-axis) is plotted against the total number of position changes (y-axis). (B) Venn diagram depicting the overlap of REGGAE results for the five different input lists.


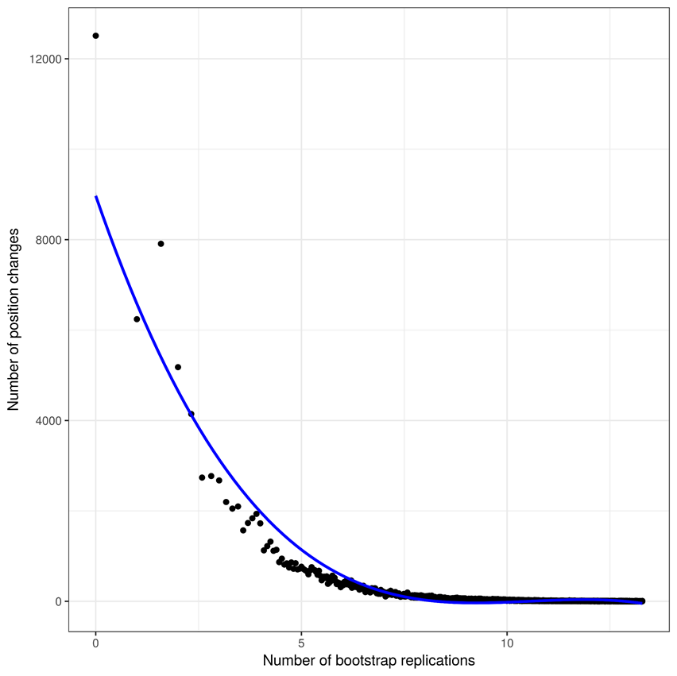

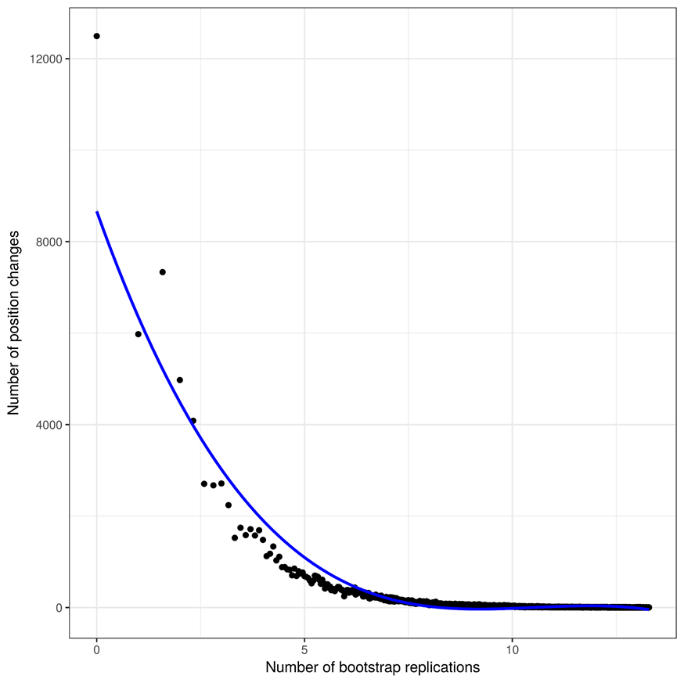


Figure 4. Effect of an increasing number of bootstrap replications on the order of the regulators in the REGGAE result lists for the top 1719 upregulated genes. The number of bootstrap samples (x-axis) is plotted against the total number of position changes (y-axis). (B) Venn diagram depicting the overlap of REGGAE results for the five different input lists.

Figure 3. Effect of an increasing number of bootstrap replications on the order of the regulators in the REGGAE result lists for the top 750 upregulated genes. The number of bootstrap samples (x-axis) is plotted against the total number of position changes (y-axis). (B) Venn diagram depicting the overlap of REGGAE results for the five different input lists.
