# Supplementary material for: REGGAE: a novel approach for the identification of key transcriptional regulators
Source: Bioinformatics. 2018 May 7;34(20):3503–10. doi: 10.1093/bioinformatics/bty372 (PMC6184769; doi:10.1093/bioinformatics/bty372)
Supplement: Supplementary Data [file bty372_suppl_data.zip › Supplement_S6_Bioinformatics.docx]

**REGGAE: a novel approach for the identification of key transcriptional regulators**

Tim Kehl^1,*^, Lara Schneider^1^, Kathrin Kattler^2^, Daniel Stöckel^1^, Jenny Wegert^3^, Nico Gerstner^1^, Nicole Ludwig^4^, Ute Distler^5^, Markus Schick^7^, Ulrich Keller^7,8^, Stefan Tenzer^5^, Manfred Gessler^3^, Jörn Walter^2^, Andreas Keller^1^, Norbert Graf^6^, Eckart Meese^4^, Hans-Peter Lenhof^1^

^1^Center for Bioinformatics, Saarland Informatics Campus, Saarland University, Saarbrücken, Germany, ^2^Department of Genetics, Saarland University, Saarbrücken, Germany, ^3^Theodor-Boveri-Institute/Biocenter, Developmental Biochemistry, and Comprehensive Cancer Center Mainfranken, Würzburg University, Würzburg, Germany, ^4^Human Genetics, Saarland University, Homburg, Germany, ^5^Institute for Immunology, Johannes Gutenberg University Mainz, Mainz, Germany, ^6^Department of Pediatric Oncology and Hematology, Medical School, Saarland University, Homburg, Germany, ^7^Internal Medicine III, School of Medicine, Technische Universität München, Munich, Germany, ^8^German Cancer Consortium (DKTK), German Cancer Research Center (DKFZ), Heidelberg, Germany

*To whom correspondence should be addressed.

# Supplement S6

In this supplement, we discuss further results for the comparison of ER-positive and ER-negative breast cancer samples. Here we compare the top 5 candidates of each method compared to all other approaches. The top candidates for REGGAE and TFRank were already discussed in the main manuscript. This is an extension of this analysis. In the following section we show the results for the different methods and describe if there are direct connections to ER-positive breast cancers or even breast cancer in general.

**CSA**

The top five candidates of CSA are UBP1, SOX10, FEV, SUMO1 and NCAPG2. Of those we find SOX10 to be expressed in tripel-negative breast cancer samples (Cimino-Mathews *et al.*, 2013) and SUMO1 to be upregulated in inflammatory breast cancer (Chai *et al.*, 2016).

**Table 1. Top 5 Regulators identified by CSA in comparison to other approaches.**

| Regulators | CSA | REGGAE | RIF1 | RIF2 | TDD | TED | TFactS | TFRank |
| --- | --- | --- | --- | --- | --- | --- | --- | --- |
| UBP1 | **9.76987e-06** | 1.0 (649) | 0.805 (627) | -0.482 (785) | **0.0004 (131)** | 1.0 (74) | **0.030 (341)** | 0.048 (851) |
| SOX10 | **9.76987e-06** | 1.0 (482) | -1.660 (404) | -2.025 (575) | **0.0003 (145)** | 1.0 (67) | 0.171 (463) | 0.035 (869) |
| FEV | **9.76987e-06** | 1.0 (395) | **-3.476 (38)** | **7.990 (22)** | 0.00018 (206) | 1.0 (267) | **2.21e-07 (161)** | 0.163 (781) |
| SUMO1 | **9.76987e-06** | 1.0 (543) | -0.054 (985) | -1.995 (536) | 0.0001 (207) | 1.0 (162) | **6.15e-05 (194)** | 0.155 (788) |
| NCAPG2 | **9.76987e-06** | 1.0 (892) | **2.612 (48)** | 0.303 (872) | **0.0004 (132)** | 0.061 (5) | **1.780e-25 (43)** | 0.183 (776) |

**RIF1**

The top five candidates of RIF1 are CISH, LRIG1, ERBB2, PRL and SOCS3. LRIG1 is known to correlate with relapse-free survival in ERα-positive breast cancer (Krig. *et al.,* 2013). ERBB2 is prominent a breast cancer marker (Slamon *et al.,* 2001). PRL was found to be downregulated in breast cancers compared to normal tissue (Hachim. *et al.,* 2001) and downregulation of SOCS3 is associated with the risk of recurrence in breast carcinoma (Ying *et al.*, 2001).

**Table 2. Top 5 Regulators identified by RIF1 in comparison to other approaches.**

| Regulators | RIF1 | CSA | REGGAE | RIF2 | TDD | TED | TFactS | TFRank |
| --- | --- | --- | --- | --- | --- | --- | --- | --- |
| CISH | **-17.716** | 1.0 (841) | 0.925 (341) | **21.764 (1)** | **1.0 (3)** | 0.060 (8) | 0.820 (637) | **3.72 (24)** |
| LRIG1 | **-13.521** | 1.0 (906) | 1.0 (366) | **7.881 (17)** | **0.333 (13)** | 0.157 (19) | 0.351 (535) | 2.71 (441) |
| ERBB2 | **-12.451** | 1.0 (879) | 1.0 (387) | **26.223 (2)** | **1.0 (6)** | 0.061 (7) | 0.484(573) | 0.001(1027) |
| PRL | **-11.846** | 1.0 (835) | 1.0 (428) | **6.537 (31)** | 0.0 (1063) | **0.050 (3)** | 0.440 (561) | 0.001(1036) |
| SOCS3 | **11.764** | 1.0 (845) | 1.0 (511) | 2.274(326) | **0.167 (17)** | 0.157 (20) | 1.0 (850) | 0.001(1029) |

**RIF2**

The top five candidates of RIF2 are CISH, ERBB2, MAP3K1, HOXD1 and GRHL1. ERBB2 is prominent a breast cancer marker (Slamon *et al.,* 2001). Mutations of MAP3K1 can be linked to the prognosis of MAP3K1 early breast cancers (Kuo *et al.,* 2017). An increased methylation of HOXD1 was found in low-grade invasive breast cancers. GRHL1 was identified as a downstream target of the ER-receptor (Zheng *et al.*, 2016).

**Table 3. Top 5 Regulators identified by RIF2 in comparison to other approaches.**

| Regulators | RIF2 | CSA | REGGAE | RIF1 | TDD | TED | TFactS | TFRank |
| --- | --- | --- | --- | --- | --- | --- | --- | --- |
| CISH | **21.764** | 1.0 (841) | 0.926 (341) | **-17.717 (1)** | **1.0 (3)** | 0.061 (8) | 0.820 (637) | **3.722 (24)** |
| ERBB2 | **26.22** | 1.0 (879) | 1.0 (387) | **-12.452 (3)** | **1.0 (6)** | 0.061 (7) | 0.484 (573) | 0.002 (1027) |
| MAP3K1 | **14.628** | 1.0 (838) | 1.0 (380) | **-3.941 (46)** | **1.0 (7)** | 0.061 (12) | 0.820 (640) | **3.670 (25)** |
| HOXD1 | **10.667** | 1.0 (846) | 1.0 (392) | **-3.120 (27)** | **0.003 (39)** | 1.0 (112) | 1.0 (923) | 0.008 (964) |
| GRHL1 | **11.399** | 1.0 (709) | **2.582e-05 (237)** | -2.561 (143) | **0.0002 (171)** | 1.0 (136) | 0.610 (607) | 0.074 (832) |

**TDD**

The top five candidates of TDD are GH1, NR0B2, CISH, DND1 and DUSP6. Mutations in GH1 were found to be associated with breast cancer development (Wagner *et al.,* 2007). DND1 promotes breast cancer apoptosis (Cheng *et al.,* 2017). DUSP6 was found to be upregulated in MCF7 breast cancer cells after treatment with phorbol ester (Nunes-Xavier *et al.*, 2010).

**Table 4. Top 5 Regulators identified by TDD in comparison to other approaches.**

| Regulators | TDD | CSA | REGGAE | RIF1 | RIF2 | TED | TFactS | TFRank |
| --- | --- | --- | --- | --- | --- | --- | --- | --- |
| GH1 | **2** | 1.0 (869) | 1.0 (441) | **-6.715 (9)** | -1.072 (556) | 0.061 (6) | 0.201 (477) | 0.002 (1011) |
| NR0B2 | **1** | 1.0 (840) | 1.0 (437) | **-4.182 (17)** | **3.517 (147)** | 0.061 (9) | 0.820 (638) | 0.004 (987) |
| CISH | **1** | 1.0 (841) | 0.926 (341) | **-17.717 (1)** | **21.764 (1)** | 0.061 (8) | 0.820 (637) | **3.722 (24)** |
| DND1 | **1** | 1.0 (839) | 1.0 (446) | **-6.503 (8)** | **-9.005 (8)** | 0.061 (10) | 0.820 (641) | 0.001 (1042) |
| DUSP6 | **1** | 1.0 (843) | 1.0 (501) | -1.034 (326) | **-9.785 (9)** | 0.061 (11) | 0.820 (639) | 0.002 (1039) |

**TED**

The top four and only significant candidates of TDD are MEIS1, UTF1, PRL and RBMS1. Of these MEIS1 was identified as favorable prognostic and predictive biomarkers for ER-positive breast cancer (Doolan *et al.*, 2012) and PRL was found to be downregulated in breast cancers compared to normal tissue (Hachim. *et al.,* 2001).

**Table 5. Top 5 Regulators identified by TDD in comparison to other approaches.**

| Regulators | TED | CSA | REGGAE | RIF1 | RIF2 | TDD | TFactS | TFRank |
| --- | --- | --- | --- | --- | --- | --- | --- | --- |
| MEIS1 | **0.001** | 1.0 (743) | 1.0 (508) | -2.008 (206) | -0.277 (700) | **0.003 (36)** | **0.0005 (227)** | 0.018 (909) |
| UTF1 | **0.049** | 1.0 (836) | 1.0 (458) | **-3.080(39)** | **3.013 (144)** | 0.0 (1285) | 0.440 (563) | 0.001 (1045) |
| PRL | **0.049** | 1.0 (835) | 1.0 (428) | **-11.846 (4)** | **6.537 (31)** | 0.0 (1063) | 0.440 (561) | 0.002 (1036) |
| RBMS1 | **0.049** | 1.0 (847) | 1.0 (542) | -1.426 (399) | **-8.167 (14)** | 0.0 (1236) | 0.440 (562) | 0.001 (1050) |

**TFactS**

The top five candidates of TFactS are KMT2C, ARNTL, CNOT3, TFAP2A and SUMO2. KMT2C was found to be mutated in the TCGA dataset (The Cancer Genome Atlas Network, 2009). ARNTL was identified as hypermethylated in promotor regions of several breast cancer tissues (Kuo *et al.*, 2009). Differential expression of alternative TFAP2A isoforms has been detected during breast tumorigenesis (Berlato et al., 2011).

**Table 6. Top 5 Regulators identified by TFactS in comparison to other approaches.**

| Regulators | TFactS | CSA | REGGAE | RIF1 | RIF2 | TDD | TED | TFRank |
| --- | --- | --- | --- | --- | --- | --- | --- | --- |
| KMT2C* | **4.90e-61** | - | - | - | - | - | 1.0 (467) | 0.815 (618) |
| ARNTL | **9.222e-59** | **9.77e-06 (65)** | 1.0 (547) | 0.569 (722) | -2.150 (454) | 1.298e-05 (463) | 1.0 (255) | 1.113 (538) |
| CNOT3 | **1.136e-49** | **9.77e-06 (167)** | **0.0002 (250)** | -0.165 (966) | -2.593 (352) | 1.153e-05 (478) | 1.0 (420) | 1.101 (554) |
| TFAP2A | **1.573e-49** | **9.77e-06 (174)** | **1.578e-73 (37)** | -1.450 (329) | -0.991 (836) | 1.074e-05 (487) | 1.0 (1011) | **1.193 (36)** |
| SUMO2 | **1.270e-48** | **9.77e-06 (298)** | **0.002 (271)** | 0.730 (937) | -1.756 (563) | 1.008e-05 (498) | 1.0 (886) | 1.139 (490) |

*For KMT2C no gene expression values were available in the used dataset.

# References

Berlato et al. (2011) Alternative TFAP2A isoforms have distinct activities in breast cancer. Breast Cancer Res. **13**. R23.

Chai *et al.* (2016) Systematically identify key genes in inflammatory and non-inflammatory breast cancer. *International Journal of Oncology*, **575**, 600-14.

Cheng *et al.* (2017) RNA-Binding Protein Dnd1 Promotes Breast Cancer Apoptosis by Stabilizing the Bim mRNA in a miR-221 *Binding Site. Biomed Res Int*. **2017**. 9596152.

Cimino-Mathews. *et al.* (2013) Neural crest transcription factor Sox10 is preferentially expressed in triple-negative and metaplastic breast carcinomas. *Human Pathology*, **44**, 959-65.

Doolan *et al.* (2012) TMEM25, REPS2 and Meis 1: favourable prognostic and predictive biomarkers for breast cancer. Tumour Biology. **30**. 200-9.

Faryna *et al.* (2012) Genome-wide methylation screen in low-grade breast cancer identifies novel epigenetically altered genes as potential biomarkers for tumor diagnosis. FASEB Journal, **26**, 4937-50.

Hachim *et al.* (2001) A favorable role of prolactin in human breast cancer reveals novel pathway-based gene signatures indicative of tumor differentiation and favorable patient outcome. Human Pathology*.* **53**, 142-52.

Krig *et al.* (2013) Lrig1 is an estrogen-regulated growth suppressor and correlates with longer relapse-free survival in ERα-positive breast cancer. *Molecular Cancer Research*, **9**, 1460-17.

Kuo *et al.* (2009) Disturbance of circadian gene expression in breast cancer. Virchows Arch. **454**. 467-74.

Kuo *et al.* (2017) Polymorphisms of ESR1, UGT1A1, HCN1, MAP3K1 and CYP2B6 are associated with the prognosis of hormone receptor-positive early breast cancer. *Oncotarget*, **8**, 20925-20938.

Nunes-Xavier *et al.* (2010) Differential Up-regulation of MAP Kinase Phosphatases MKP3/DUSP6 and DUSP5 by Ets2 and c-Jun Converge in the Control of the Growth Arrest Versus Proliferation Response of MCF-7 Breast Cancer Cells to Phorbol Ester. *J Biol Chem*. **285**. 26417-30

Slamon *et al.* (2001) Use of Chemotherapy plus a Monoclonal Antibody against HER2 for Metastatic Breast Cancer That Overexpresses HER2. *The New England Journal of Medicine.* **344**, 783-792.

The Cancer Genome Atlas Network (2009). Comprehensive molecular portraits of human breast tumors. Nature. **490**. 61-70.

Wagner *et al.* (2007) The GH1/IGF-1 axis polymorphisms and their impact on breast cancer development. Breast Cancer Res Treat. **104**. 233-48.

Ying *et al.* (2010) Loss of SOCS3 expression is associated with an increased risk of recurrent disease in breast carcinoma. *Journal of Cancer Research and Clinical Oncology.* **136**, 1617–1626.

Zheng *et al.* (2016) Role of estrogen receptor in breast cancer cell gene expression. *Molecular Medicine Reports*.13(5):4046-50.
