# Supplementary material for: REGGAE: a novel approach for the identification of key transcriptional regulators
Source: Bioinformatics. 2018 May 7;34(20):3503–10. doi: 10.1093/bioinformatics/bty372 (PMC6184769; doi:10.1093/bioinformatics/bty372)
Supplement: Supplementary Data [file bty372_suppl_data.zip › Supplement_S7_Bioinformatics.docx]

**REGGAE: a novel approach for the identification of key transcriptional regulators**

Tim Kehl^1,*^, Lara Schneider^1^, Kathrin Kattler^2^, Daniel Stöckel^1^, Jenny Wegert^3^, Nico Gerstner^1^, Nicole Ludwig^4^, Ute Distler^5^, Markus Schick^7^, Ulrich Keller^7,8^, Stefan Tenzer^5^, Manfred Gessler^3^, Jörn Walter^2^, Andreas Keller^1^, Norbert Graf^6^, Eckart Meese^4^, Hans-Peter Lenhof^1^

^1^Center for Bioinformatics, Saarland Informatics Campus, Saarland University, Saarbrücken, Germany, ^2^Department of Genetics, Saarland University, Saarbrücken, Germany, ^3^Theodor-Boveri-Institute/Biocenter, Developmental Biochemistry, and Comprehensive Cancer Center Mainfranken, Würzburg University, Würzburg, Germany, ^4^Human Genetics, Saarland University, Homburg, Germany, ^5^Institute for Immunology, Johannes Gutenberg University Mainz, Mainz, Germany, ^6^Department of Pediatric Oncology and Hematology, Medical School, Saarland University, Homburg, Germany, ^7^Internal Medicine III, School of Medicine, Technische Universität München, Munich, Germany, ^8^German Cancer Consortium (DKTK), German Cancer Research Center (DKFZ), Heidelberg, Germany

*To whom correspondence should be addressed.

Supplement S7

In the following sections, we describe the analyses for the MYC permutation signatures (GSE7897).

## General information

Gene expression differences were calculated using the shrinkage t-test proposed by Opgen-Rhein and Strimmer (Opgen-Rhein and Strimmer, 2007). We then selected the top 250 up- and downregulated genes. For each analysis, we used the entire collection of mouse regulator-target interactions (RTIs) from the RegulatorTrail web service (Kehl *et al.*, 2017) (Version 2).

## Individual algorithms

Parameters for all analyses are described in the following sections. Corresponding results can be found in Supplement S6.

### Correlation set analysis (CSA)

For the Correlation set analysis (Huang *et al.*, 2012), we used the implementation provided by the RegulatorTrail web service. For the lists containing upregulated genes, we calculated an upper-tailed p-value and for downregulated genes a lower-tailed one. All p-values were estimated using a permutation test with 1,000,000 random permutations and an additional pseudo-count.

### REGGAE

For the REGGAE analysis, we used the implementation provided by the RegulatorTrail web service. We sorted the list of upregulated genes decreasingly and the list of downregulated genes increasingly. As described in Section 2.3.2 in the main manuscript, we first computed Pearson’s correlation coefficients between all genes and all associated regulators, and based on this information, we built the associated regulator lists. For upregulated genes, we sorted association scores decreasingly and for downregulated genes increasingly. Finally, we performed enrichment analysis using the Wilcoxon rank-sum test to detect the most influential regulators. All REGGAE analyses were performed using 1,000 random bootstrap replications.

### RIF1 and RIF2

For the RIF1 and RIF2 analysis (Reverter *et al.*, 2010), we used the implementation provided by RegulatorTrail. We used Pearson’s correlation coefficients to compute the differences in correlation (between the two groups of interest) for each regulator and its target genes in the analyzed gene lists and the fold-change to calculate differential expression.

### TDD

For the TDD analysis (Yang *et al.*, 2013), we implemented a Python script that calculates the respective statistic.

### TED

In order to perform the analysis proposed by Yang et al. (Yang *et al.*, 2013), we used the Binomial test implemented in the RegulatorTrail web service. As a reference set we used all genes that are targeted by a regulator in the used collection of RTIs. The resulting p-values are adjusted using the Benjamini and Yekutieli method (Benjamini and Yekutieli, 2001).

### TFactS

In order to perform the analysis proposed by Essaghir et al. (Essaghir *et al.*, 2010), we used the Hypergeometric test implemented in the RegulatorTrail web service. As a reference set we used all genes that are targeted by a regulator in the used collection of RTIs. The resulting p-values are adjusted using the Benjamini and Yekutieli method (Benjamini and Yekutieli, 2001).

### TFRank

For the TFRank analysis (Goncalves *et al.*, 2011), we used the prototype implementation provided on the authors' web site (<http://web.tecnico.ulisboa.pt/aplf/code/tfrank/>). We used the unweighted network given by our collection of RTIs and the standard parameters also provided on the authors' web site.

### TFRank

For the TFRank analysis (Goncalves *et al.*, 2011), we used the prototype implementation provided on the authors' web site (<http://web.tecnico.ulisboa.pt/aplf/code/tfrank/>). We used the unweighted network given by our collection of RTIs and the standard parameters also provided on the authors' web site.

References

Benjamini,Y. and Yekutieli,D. (2001) The control of the false discovery rate in multiple testing under dependency. *Annals of statistics*.

Essaghir,A. *et al.* (2010) Transcription factor regulation can be accurately predicted from the presence of target gene signatures in microarray gene expression data. *Nucleic Acids Research*, **38**, e120–e120.

Goncalves,J.P. *et al.* (2011) TFRank: network-based prioritization of regulatory associations underlying transcriptional responses. *Bioinformatics*, **27**, 3149–3157.

Huang,C.-L. *et al.* (2012) Correlation set analysis: detecting active regulators in disease populations using prior causal knowledge. *BMC Bioinformatics*, **13**, 46.

Kehl,T. *et al.* (2017) RegulatorTrail: a web service for the identification of key transcriptional regulators. *Nucleic Acids Research*, **45**, W146–W153.

Opgen-Rhein,R. and Strimmer,K. (2007) Accurate Ranking of Differentially Expressed Genes by a Distribution-Free Shrinkage Approach. *Statistical Applications in Genetics and Molecular Biology*, **6**.

Reverter,A. *et al.* (2010) Regulatory impact factors: unraveling the transcriptional regulation of complex traits from expression data. *Bioinformatics*, **26**, 896–904.

Yang,J. *et al.* (2013) DCGL v2.0: An R Package for Unveiling Differential Regulation from Differential Co-expression. *PLOS ONE*, **8**, e79729.
